# Predictors of stress resilience in Parkinson’s disease and associations with symptom progression

**DOI:** 10.1038/s41531-024-00692-4

**Published:** 2024-04-11

**Authors:** Anouk van der Heide, Lisanne J. Dommershuijsen, Lara M. C. Puhlmann, Raffael Kalisch, Bastiaan R. Bloem, Anne E. M. Speckens, Rick C. Helmich

**Affiliations:** 1 Radboud University Medical Centre, Department of Neurology, Centre of Expertise for Parkinson & Movement Disorders, Nijmegen, the Netherlands; 2 Radboud University, Donders Institute for Brain Cognition and Behavior, Centre for Cognitive Neuroimaging, Nijmegen, the Netherlands; 3 Leibniz Institute for Resilience Research, Mainz, Germany; 4 Neuroimaging Center, Focus Program Translational Neuroscience, Johannes Gutenberg University Medical Center, Mainz, Germany; 5 Radboud University Medical Centre, Department of Psychiatry, Nijmegen, the Netherlands

**Keywords:** Parkinson's disease, Epidemiology, Risk factors, Predictive markers

## Abstract

People with Parkinson’s disease (PD) are sensitive to effects of long-term stress, but might differ in stress resilience, i.e. the ability to maintain mental health despite adversity. It is unclear whether stress resilience in PD is predominantly determined by dopamine deficiency, psychosocial factors, or both. In PD animal models, chronic stressors accelerate disease progression, but evidence in humans is lacking. Our objectives were to (1) distinguish stressor-reactive from resilient PD patients, (2) identify resilience factors, and (3) compare symptom progression between stressor-reactive and resilient patients. We conducted a longitudinal survey in Personalized Parkinson Project participants (*N* = 350 PD). We used the COVID-19 pandemic as a model of a stressor, aligned in time for the entire cohort. COVID-19-related stressors, perceived stress, and PD symptoms were assessed at 11 timepoints (April-October 2020). Both pre-COVID and in-COVID clinical assessments were available. We quantified stressor-reactivity as the residual between actual and predicted perceived stress relative to COVID-19-related stressors, and modeled trajectories of stressor-reactivity across timepoints. We explored pre-COVID predictors of 6-month average stressor-reactivity, and tested whether stressor-reactivity was prospectively associated with one-year clinical progression rates. Latent class trajectory models distinguished patients with high (*N* = 123) or low (*N* = 227) stressor-reactivity. Pre-existing anxiety, rumination and non-motor symptom severity predicted high stressor-reactivity (risk factors), whereas quality of life, social support, positive appraisal style and cognitive abilities predicted low stressor-reactivity (resilience factors). PD-specific factors, e.g. disease duration, motor severity, and levodopa use, did not predict stressor-reactivity. The COVID-19 pandemic did not accelerate disease progression, but worsened depressive symptoms in stressor-reactive PD patients.

## Introduction

It is well-known that individuals with Parkinson’s disease (PD) are sensitive to the effects of stress^1^. Many individuals experience stress-related neuropsychiatric symptoms such as depression or anxiety^2^. Furthermore, under stressful conditions, PD motor symptoms temporarily worsen and dopaminergic medication is less effective^3,4^. One explanation for this increased stress-sensitivity is that striatal dopamine release is needed to respond adequately to stressful events^5^, for example by flexibly implementing behavioral repertoires^6^. The low levels of dopamine that are typical in PD might be insufficient to adequately respond to stressor exposure. Furthermore, dopamine depletion in the cortico-striatal reward circuitry has been linked to stress-related symptoms in PD, such as depression^7^. Besides immediate effects of acute stress, chronic stress might have detrimental long-term effects^8^. Animal models even suggest that it accelerates disease progression^9,10^, but this is not yet confirmed in humans. A better understanding of factors contributing to stressor-reactivity in PD may help predict who could be at-risk of developing neuropsychiatric complaints after stressful events. Furthermore, it is important to better understand the effects of psychological stress on the PD disease course, as a potential modifiable target for treatment.

Stress research consistently finds that not everybody reacts the same to adversity, or develops mental health problems even after chronic stressor exposure^11–13^. Resilience has been defined as maintenance or quick recovery of mental health during and after periods of adversity^11^. This can partly be predicted by personal and contextual factors, termed resilience factors, that facilitate coping with stressful situations^12,14^. Resilience factors, especially if they are malleable, are interesting targets for interventions aiming at preventing stress-related problems in at-risk individuals^15,16^. However, knowledge about resilience in PD, and its possible impact on symptoms and disease progression, is largely lacking.

Stressor-reactivity and resilience can only be studied properly in the context of stressful circumstances, ideally in combination with knowledge about functioning prior to the adversity^11^. In this study, we used the COVID-19 pandemic as natural experiment^17^. We conceptualized stressor-reactivity (SR) as the degree to which an individual’s mental health responds to COVID-19 stressors at a specific time. We regarded individuals with consistently low SR scores over an extended period of time (here, six months) as more resilient, in line with previous research^16,18^. Governmental measures to prevent spread of SARS-CoV-2, including lockdowns, social distancing and sanitation rules, together with fear of infection, caused significant psychological distress, especially in groups with pre-existing health issues^19,20^. We administered a longitudinal survey (at eleven timepoints between April-October 2020) in an existing PD cohort where pre-COVID clinical data were available (Personalized Parkinson Project, PPP^21^). We aimed to (1) distinguish stressor-reactive from resilient patients, (2) explore predictors for stressor-reactivity in PD, and (3) explore the effect of the COVID-19 pandemic and stressor-reactivity on PD symptom progression.

## Results

### Participant characteristics

**Fig. 1:**
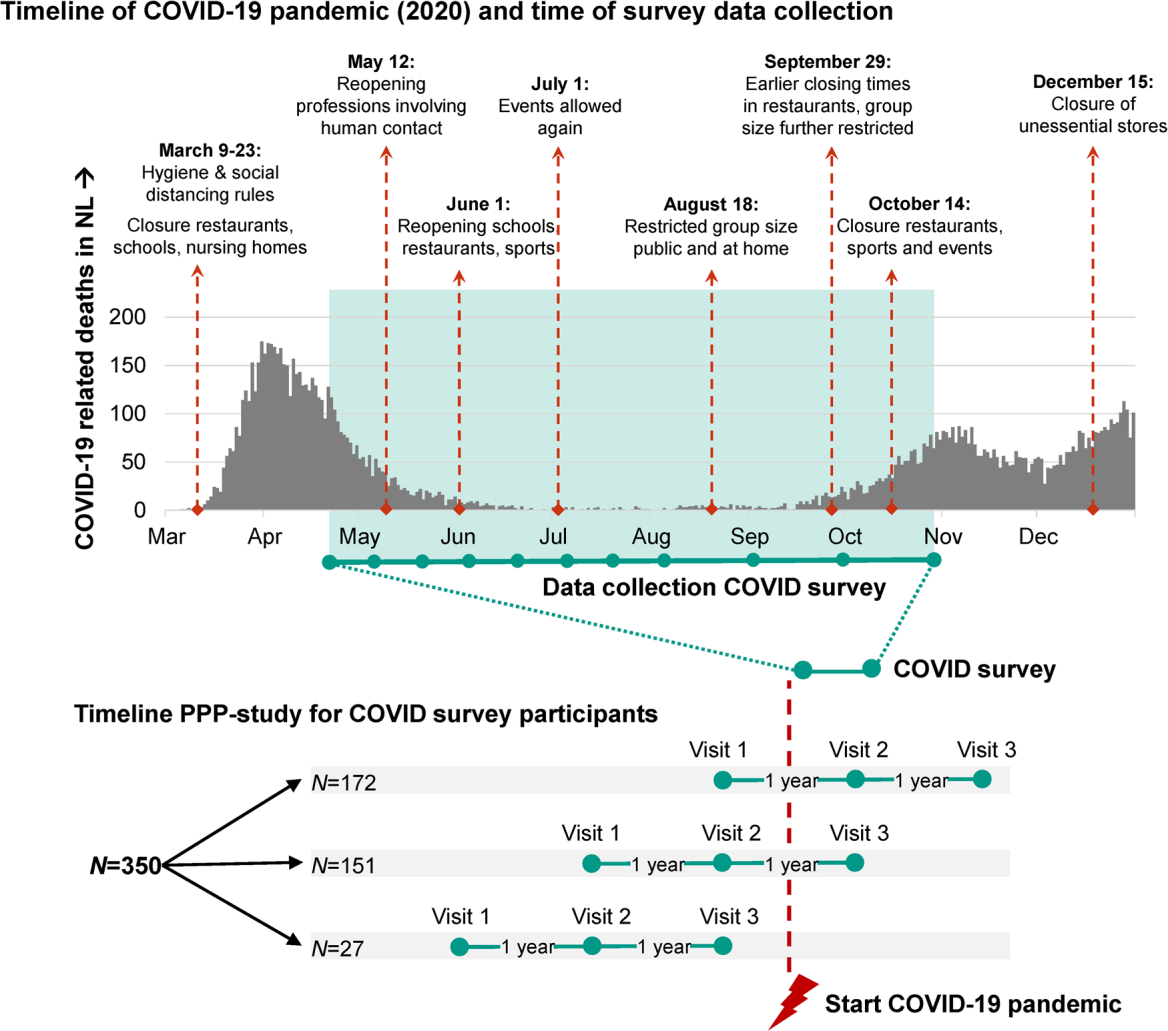
Timeline of COVID-19 pandemic and survey data collection. Overview of COVID-19-related deaths and government measures in the Netherlands to prevent spread of COVID-19 (March-December 2020) and the survey time-window (green) within the clinical PPP study.

In total, 350 participants were included (38.4% women), with a mean (SD) age of 62.7 (9.0) years at the baseline survey. The mean PD disease duration was 3.8 (1.6) years. For all participants, clinical data (motor, cognitive and psychological tests) were available that were collected during the annual PPP study visits (for an overview, see Methods section). Further characteristics are shown in Table 1. For 172 participants, clinical data were available from one pre-COVID and two in-COVID PPP visits, for 151 participants from two pre-COVID and one in-COVID PPP visit, and 27 participants had all PPP visits pre-COVID (Fig. 1). There was one year between all visits. Participants responded to a median (IQR) of 11 (10-11) COVID-surveys (same for high and low SR groups).

**Table 1:** Demographics of the study population

Variable	Survey sample (*N* = 350)					*N* valid
Age, years (SD)	62.8 (9.0)					350
Sex, *N* (% women)	134 (38.3%)					350
Education, years (SD)	17.4 (4.0)					350
Living situation, N (%)						350
With partner	226 (64.6%)					
With family	90 (25.7%)					
Alone	34 (9.7%)					
Main daily activity, *N* (%)						350
Retired or pre-retirement	143 (40.9%)					
Paid job	107 (30.6%)					
No paid work						
Voluntary work	22 (6.3%)					
No paid work due to illness	56 (16.0%)					
Not working						
Household duties	19 (5.4%)					
Involuntarily no job	3 (0.9%)					
PD duration, years (SD)	3.7 (1.7)					350
Use of levodopa/dopamine agonist, *N* (%)	330 (94.3%)					350
Levodopa equivalent dose, mg/day (SD)	563.8 (368.6)					348
MDS-UPDRS-Ia, total (SD)	2.6 (2.4)					349
MDS-UPDRS-Ib, total (SD)	9.4 (3.9)					332
MDS-UPDRS-II, total (SD)	8.3 (5.9)					332
MDS-UPDRS-III, total (SD)						343
OFF PD medication	34.8 (12.8)					
ON PD medication (*N* = 330)	29.6 (12.5)					
Hoehn & Yahr (HY) stage, *N*	HY 0	HY 1	HY 2	HY 3	HY 4	347
OFF PD medication	-	13	300	29	5	
ON PD medication (*N* = 330)	1	12	306	7	4	
Beck Depression Inventory II, total (SD)	9.0 (6.3)					332
State Trait Anxiety Inventory, total (SD)						332
State anxiety	35.4 (9.1)					
Trait anxiety	35.3 (9.5)					
Parkinson Disease Questionnaire-39, SI (SD)	19.5 (10.9)					328
Montreal Cognitive Assessment, total (SD)	26.7 (2.4)					349

This table shows demographic and clinical characteristics for all included participants. Age and PD disease duration are at the time of the first COVID-19 survey, other characteristics are assessed during the last pre-COVID-19 PPP study visit. *SD* standard deviation, *SI* summary index.

### Individual stressor-reactivity

**Fig. 2:**
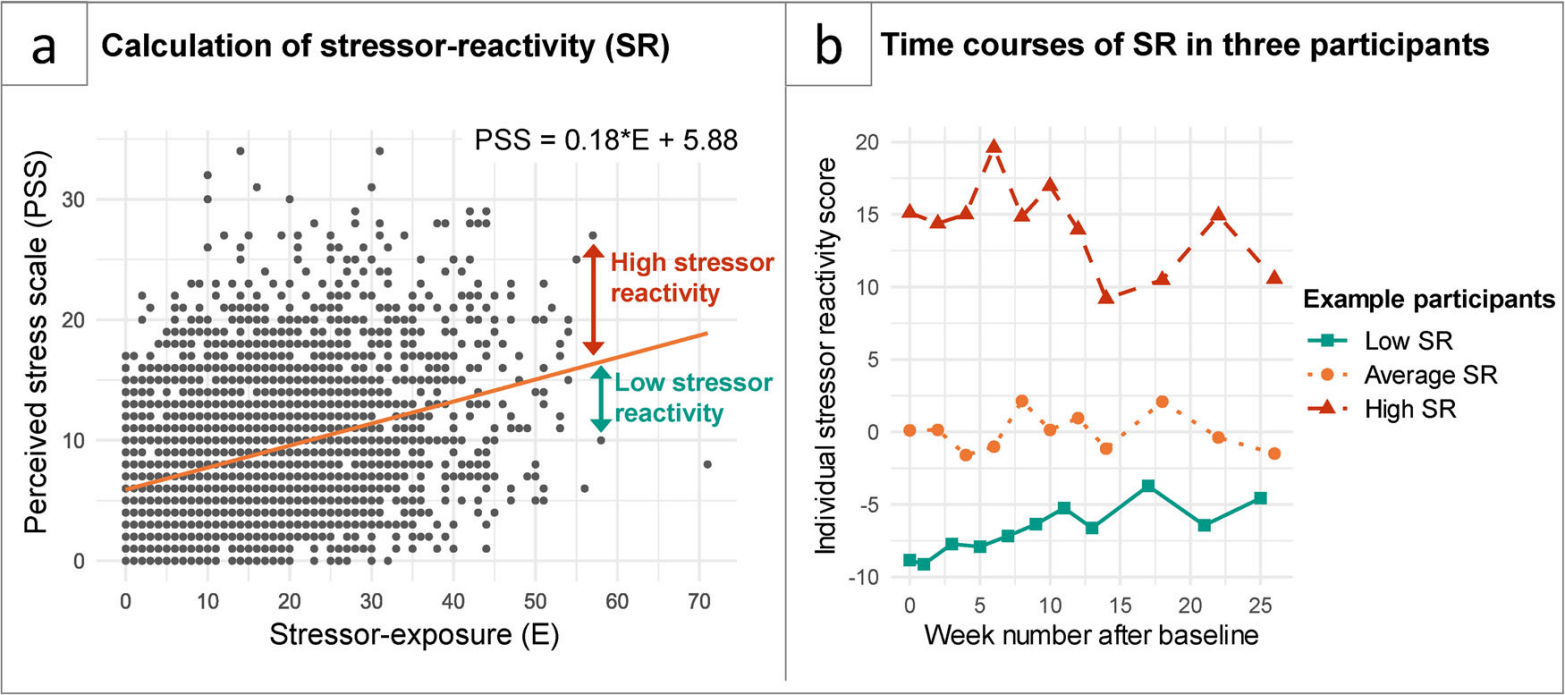
Calculation of stressor-reactivity score. **a** Visualizes the regression line between perceived stress and COVID-19-related stressor exposure. Residuals are deviations of participants from the average PSS-SL relationship. **b** Shows stressor-reactivity over time for three representative participants with stable high (red), average (orange) and low (green) stressor-reactivity. A participant with a trajectory similar to the green example can be considered comparatively resilient.

We quantified stressor-reactivity (SR), or how strongly an individual’s mental health reacted to COVID-19 stressors, by linearly regressing perceived stress (indicating stress-related problems) on stressor exposure across all participants and timepoints. For every timepoint, we determined individual stressor-reactivity scores, i.e. to what extent every participant reacted more (positive SR) or less (negative SR) to stressor exposure than predicted based on the average^16,22^ (Fig. 2a; details in the Methods section). This resulted in up to eleven scores per individual over the survey period, reflecting SR over time. Three example participants are shown in Fig. 2b. Someone with low reactivity over longer times can be classified as comparatively resilient^18^.

### Trajectories of longitudinal variables

**Fig. 3:**
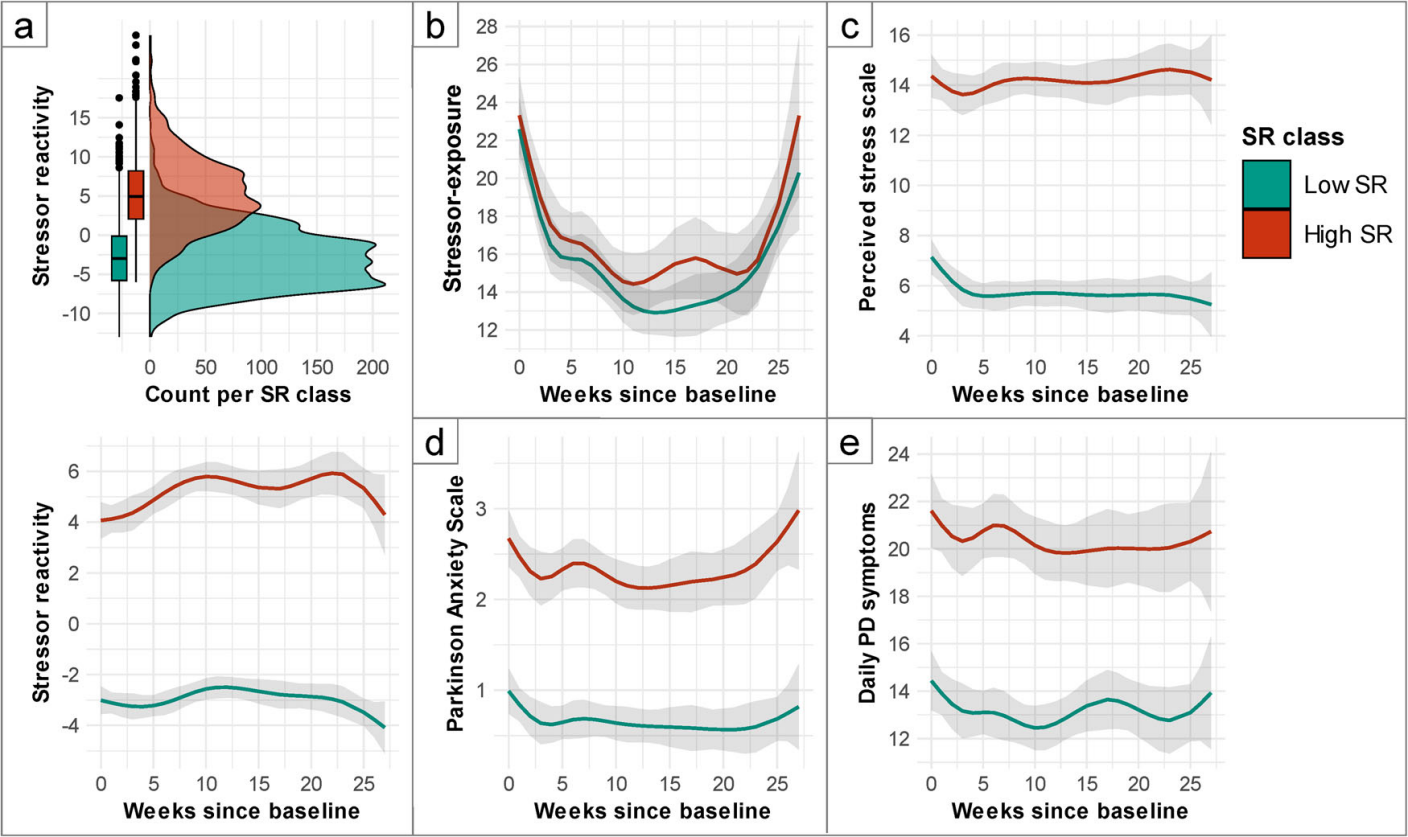
Mean trajectories and 95%-CI of longitudinal variables during the COVID-19 pandemic per stressor-reactivity class. **a** shows density plots of the SR latent classes. Model-predicted trajectories per class are plotted for stressor reactivity, (**b**) stressor exposure, (**c**) perceived stress, (**d**) episodic anxiety, and (**e**) PD symptoms (MDS-UPDRS-self), for a man of mean age (62.8), living with partner. Error bars represent 95% confidence intervals for the predicted values.

The favored latent class SR trajectory model divided the sample into one class with high (*N* = 123) and one with low stressor-reactivity (*N* = 227) (Fig. 3a), where SR remained stable during follow-up in both groups. The posterior probability of assignment (APPA) for both classes was 72% and 79%, indicating good discrimination. The trajectory of the low-SR class is compliant with a resilient response profile. Trajectories of COVID-19-related stressor exposure, stress-related problems as indexed via perceived stress (PSS), episodic anxiety (PAS) and PD symptoms (MDS-UPDRS-self) are visualized in Fig. 3b-e, with separate lines for both classes. Stressor exposure varied greatly, clearly following the pattern of COVID-19-related deaths within that period: a sharp decrease during the first six weeks, when restrictions were lifted after the first COVID-wave, and an increase when the second COVID-wave began (Fig. 3b). This did not differ between the high- and low-SR classes, suggesting absence of reporting bias. Stress-related problems decreased with decreasing stressor exposure in the first weeks, but did not increase during the second COVID-wave (Fig. 3c). Accordingly, SR (Fig. 3a) decreased towards the end of the study period rather than simply mirroring the mental health problem trajectories, which demonstrates the unique information contained by SR trajectories. Episodic anxiety levels strongly differed between classes, and globally followed the pattern of COVID-19-related deaths in both classes (Fig. 3d). Strikingly, participants with low SR reported fewer PD symptoms; the temporal profile was again similar to stressor exposure and anxiety profiles (Fig. 3e).

### Predictors of stressor-reactivity

**Fig. 4:**
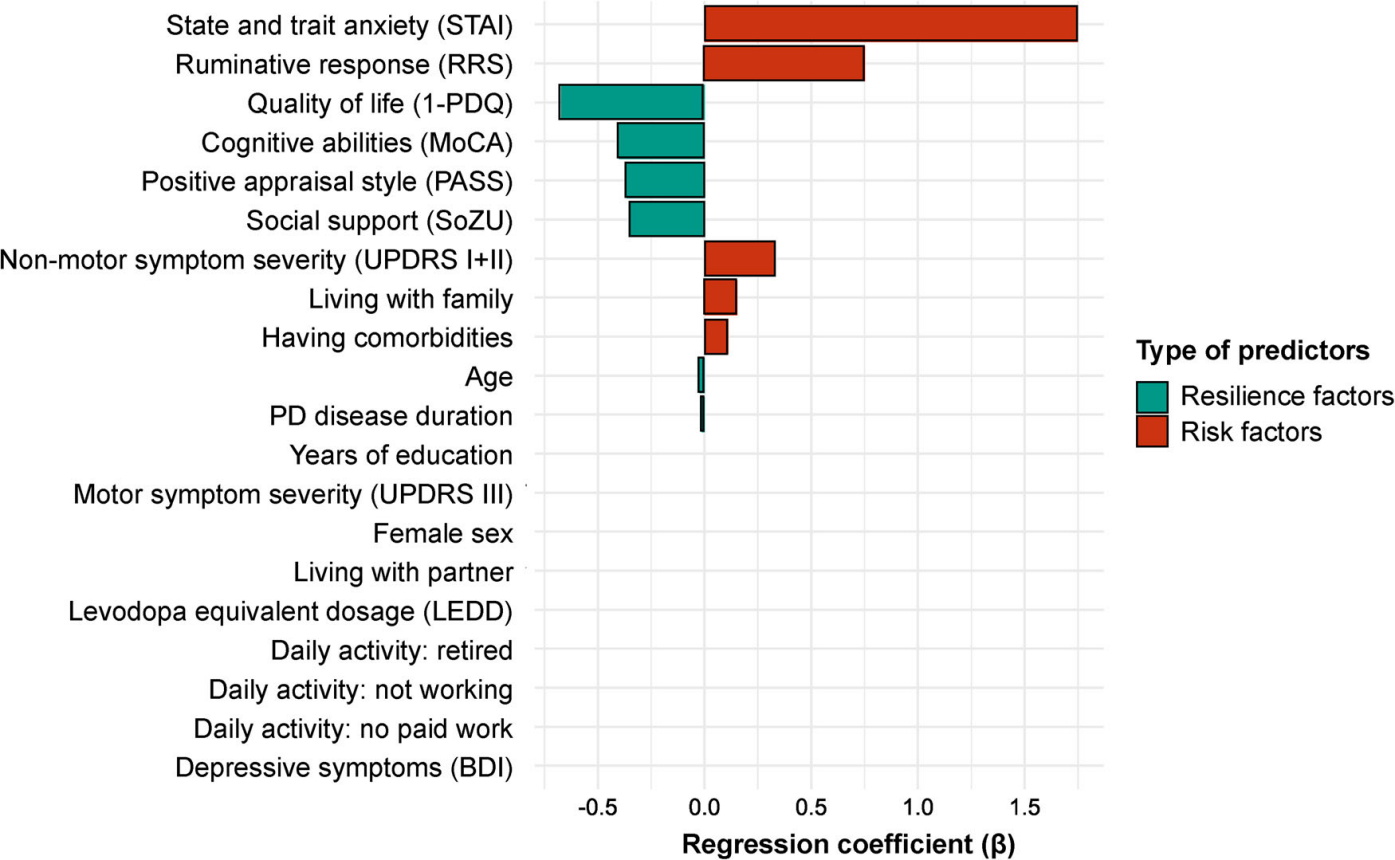
Resilience factors and risk factors for stressor-reactivity. This figure shows the β-coefficients of predictors representing the contribution of each predictor to the optimal fit. Resilience factors (negatively predicting stressor-reactivity) are shown in green, risk factors (positively predicting stressor-reactivity) in red.

The temporal changes in SR scores for the two SR classes were negligible compared to the difference in absolute average SR levels between these classes. This was confirmed by inspection of SR density plots in Fig. 3a. Rather than logistically predicting class membership, we therefore used the average SR across timepoints as dependent variable for our elastic net regression (see Methods section), to allow a more fine-grained distinction of individual SR. In the final model (R^2^ = 0.35, *p* < 0.001), the strongest negative predictors for SR, qualifying as resilience factors, were pre-pandemic quality of life (1-PDQ; regression coefficient (β) = -0.68) and cognitive abilities (MoCA; β = -0.42), as well as social support (SOZU; β = -0.36) and positive appraisal style (PASS; β = -0.37) at pandemic onset (Fig. 4 and Supplementary Table 1). In a follow-up analysis, the prediction of SR via perceived social support was partially mediated by positive appraisal style (estimated effect: -0.45 (standardized beta), *p* < 0.001; 95%-CI: -0.73 to -0.22 at alpha = 0.001; Supplementary Note 1). Strongest positive predictors, qualifying as risk factors, were pre-pandemic anxiety (STAI; β = 1.75) and non-motor symptom severity (MDS-UPDRS-self); β = 0.34), and ruminative thoughts at pandemic onset (RRS; β = 0.76).

### Associations with symptom progression

**Fig. 5:**
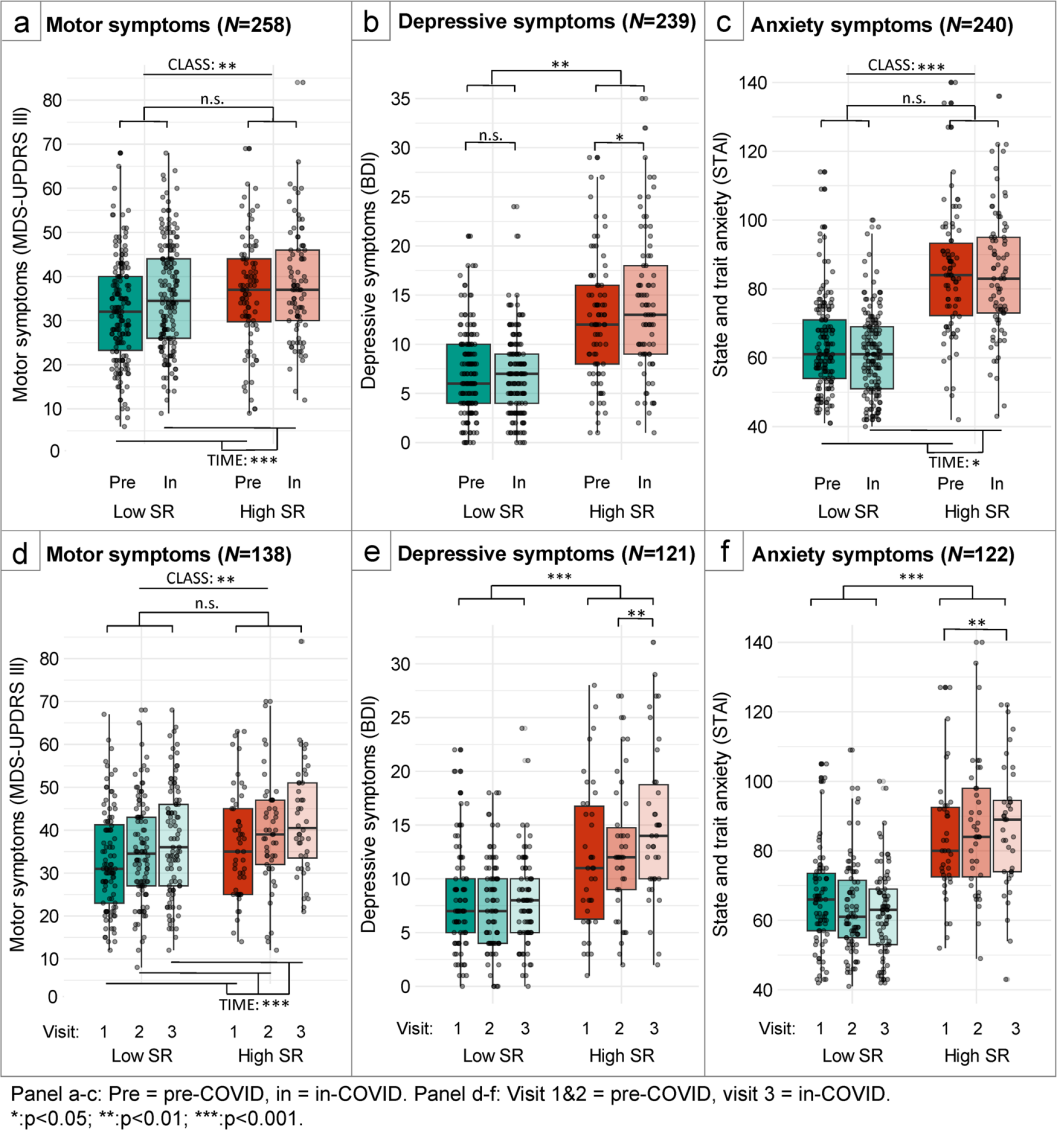
Effect of the COVID-19 pandemic on PD per SR class. Panels **a–c**: Boxplots showing symptom severity (y-axis) as a function of time (x-axis, last pre-COVID and first in-COVID assessments; one-year interval), separately for patients with low (green) and high (red) stressor-reactivity. Panels **d–f**: subgroup where two pre-COVID assessments (visit 1 and 2; one-year interval) were available, and one in-COVID assessment (visit 3). Boxes correspond to 75% of responses, tails to the remaining 25% (except outliers), centre lines correspond to median scores. Individual participants are illustrated with grey dots.

MDS-UPDRS-III scores were generally higher in patients with high SR compared to low SR scores (main effect of CLASS: F(1,251) = 6.7, *p* = 0.01). Motor symptoms generally worsened over the course of one year in both groups (main effect of TIME: F(1,255) = 26.1, *p* < 0.001) (Fig. 5a). However, the speed of motor progression was not different between patients with low and high SR (no TIME*CLASS interaction). In a subgroup (*N* = 138) with complete data for two pre-COVID and one in-COVID visits, we compared one-year progression *before* the COVID pandemic with one-year progression *during* COVID. We found similar effects as in the entire sample: no CLASS*TIME interaction, but significant main effects of TIME (F(2,284) = 18.9, *p* < 0.001) and CLASS (F(1,143) = 7.9, *p* = 0.006) (Fig. 5d).

Beck’s depressive inventory (BDI) scores increased during the COVID-19 pandemic, but only in the group with high SR (TIME*CLASS interaction: (F(1,237) = 7.8; *p* = 0.006; main effect of TIME for high SR: F(1,80) = 5.6; *p* = 0.02; Fig. 5b). The same pattern was seen in a subgroup of 123 patients with two pre-COVID and one in-COVID data points (CLASS*TIME interaction: F(2,238) = 8.6; *p* < 0.001; main effect of TIME for high SR: F(1.66,61.6) = 6.7; *p* = 0.004, no main effect of TIME for low SR (F(2,164) = 0.7; *p* = 0.51). Crucially, in the group with high SR, BDI scores increased significantly with 1.8 points (SD = 7.1) on average in the period between visits 2 and 3 (in-COVID: t(37) = -2.9, *p* = 0.02), but not between visits 1 and 2 (pre-COVID: t(37) = -0.9, *p* = 1.0) (Fig. 5e). This demonstrates that increased depressive scores were not only specific to the group with higher SR, but also specific to the COVID period.

STAI scores were higher for people with high SR than for people with low SR (main effect of CLASS: F(1,235) = 143.7; *p* < 0.001, average STAI: 84.4 ± 17.9 versus 62.1 ± 12.9 points). There was also a small but significant *decrease* in anxiety from pre-COVID to in-COVID (main effect of TIME: F(1,238) = 4.0; *p* = 0.047), but no CLASS*TIME interaction (Fig. 5c). In the subgroup of 122 patients with two pre-COVID data points however, the CLASS*TIME interaction was significant: F(2,240) = 6.3; *p* = 0.007. In the high SR group, STAI scores decreased, but only between visit 1 and 3 (delta = 3.14 ± 7.05 points, t(82) = 3.1, *p* = 0.02) (Fig. 5f).

## Discussion

We investigated resilience factors and risk factors for stressor-reactivity during the COVID-19 pandemic in PD, and tested if stressor-reactivity was associated with different symptom progression rates. We took advantage of a unique longitudinal PD cohort where we had detailed pre-COVID and in-COVID assessments available. There are three main findings. First, we observed clear subgroups of patients with low and high SR, where low SR represented more resilient patients. SR was a stable feature during follow-up. Second, important risk factors for higher SR were pre-existent anxiety, non-motor symptom severity, and rumination, whereas quality of life, cognitive abilities, social support and a positive appraisal style were resilience factors. Third, we found that high SR was not associated with a faster progression of motor symptoms during the pandemic, but related to an increase in depressive symptoms.

When starting this survey, we did not realize that COVID-19 would become the largest pandemic in modern history. This provided us with a unique opportunity to investigate the psychological impact of a major adverse situation in people with PD. The pandemic affected everyone directly or indirectly, either due to (fear of) infection, or due to governmental measures and their impact. Uncertainty and social distancing measures increased the risk of psychological distress in vulnerable people. A survey in 9,565 people from 78 countries showed that the pandemic was at least moderately stressful for most people^23^. Furthermore, a longitudinal cohort study in 5,146 elderly adults reported increased anxiety and depression (June-December 2020)^24^, underlining the impact of the COVID-19 pandemic in the elderly.

Studies about resilience in PD are scarce^25,26^ and mostly limited by cross-sectional designs, which might lead to overestimated associations. With the current design, we could explore whether reactivity to COVID-19-related stress and other longitudinal measures changed during a six-month period^16^. Stressor exposure clearly followed changes in COVID-19-related deaths. In contrast, the degree to which people perceived stress in response to these stressors was more stable. It was therefore no surprise that all latent class models divided the sample into a stable low-SR and high-SR group. Considering recent conceptualizations of resilience as a process^11^, our data suggests that malleable aspects of resilience may change slowly, over years rather than months, and there may be a strong trait-like component of resilience. Episodic anxiety and PD symptoms were high at the pandemic onset, but decreased rapidly in the first few weeks, in line with other findings^27^, likely because people adapted to the circumstances. Anxiety increased again in the high-SR PD group towards the second COVID-wave, suggesting that this group adapted less and remained vulnerable.

Given the lack of prior knowledge about resilience factors in PD, this study had an exploratory approach. The identification of predictors of stressor-reactivity in PD could ultimately inform us about the (psychological and PD-related) mechanisms that are involved. The reported predictors are a starting point for future studies, to validate whether factors that were important during the pandemic, also apply to different stressful situations. In contrast to the observation that SR remained stable during follow-up, several reported risk and resilience factors do not suggest that resilience is a stable phenotype (e.g. social support and cognitive abilities). Namely, social support was identified as an important resilience factor, in line with a large meta-analysis in the general population^28^, and consistent with the observation that people with low social support are more likely to develop depression and anxiety^29^. Especially during uncertainty, social connections can play a crucial role in limiting psychological suffering, thereby attributing to resilience^30^. Counterintuitively, living with family was a weak risk factor for SR, whereas older age seemed protective, a common finding for resilience during the pandemic^18^. A possible explanation is a previous observation that older adults experience fewer interpersonal stressors, and respond less to them, whereas for people living with family the opposite is true^31^. Another resilience factor was cognitive abilities, in line with previous work^32^. Cognitive strategies may help individuals to adapt to changing circumstances, thereby contributing to resilience. These factors imply that resilience is not stable on the long term, since social support and cognitive abilities are not personality traits and may grow or decline over time. This would imply that resilience is malleable and can possibly be trained.

Not surprisingly, high levels of anxiety and rumination were strong risk factors for SR, whereas positive appraisal style was an important resilience factor. Cognitive therapies or mindfulness interventions, which have previously been shown to lower the impact of stress and rumination^33^, should thus be considered to promote resilience. In contrast, depressive symptoms did not predict SR. This might be partly explained by the fact that BDI-scores in our sample were overall relatively low (9 points on average). During their last pre-COVID visit, only 48 of the 350 participants (14%) scored above the 14-point diagnostic cut-off for depression in PD^34^. This low number compared to the general PD population (30–40%)^2^ may be explained by a lower motivation in depressed people to participate in demanding longitudinal studies. Contrary to our findings, one study found a strong relationship between low depressive symptoms and resilience in the elderly, although this study measured resilience retrospectively^35^. Future studies may look into this discrepancy further.

Interestingly, motor symptoms (MDS-UPDRS-III) and dopaminergic medication (LEDD) did not predict SR, whereas non-motor symptoms (MDS-UPDRS-self) and the presence of comorbidities did. Disease duration was weakly associated to lower SR. Accordingly, another study showed that resilience in PD was not associated with disease severity (MDS-UPDRS), but moderately associated with less disability and better quality of life^25^. This might suggest that reduced resilience in PD is not so much related to reduced dopamine levels in the motor striatum (which correlate with motor symptoms), but instead relates to more diffuse pathology^36^ (i.e. non-motor symptoms and cognitive impairment) and generic factors (social support and psychological factors).

Contrary to our hypothesis, PD motor progression (MDS-UPDRS-III) was not accelerated during the pandemic, and the disease course was not faster in patients with high SR. This suggests that PD motor progression and perceived stress are unrelated. This conclusion would be in line with a recent study using self-rated resilience, which reported no effect of the COVID-19 pandemic on motor or cognitive trajectories in PD^37^. In contrast, another longitudinal study exploring the effects of the COVID-19 pandemic on PD disease progression showed worsening of motor and non-motor symptoms after a six-month follow-up (*N* = 33), compared to a group of PD patients measured pre-COVID (*N* = 17)^38^. Our study was considerably larger, and has the advantage of having within-subject longitudinal assessments both before and during the COVID-19 pandemic. In an attempt to explain our negative finding, we should keep in mind that effects on motor progression may arise after a longer time period than the current one-year follow-up. Another possibility is that MDS-UPDRS-III is not be sensitive enough to monitor individual changes in symptom severity, since it provides a snapshot, which can be distorted by short-term effects that are irrelevant to disease progression (such as acute stress or time of the day)^39^.

A relevant finding is that depressive symptoms of PD patients with high SR increased during the pandemic, whereas scores remained stable for people with low SR. The same was recently reported by others^37^. In contrast, anxiety during the pandemic slightly *decreased* compared to pre-pandemic scores, both for low and high SR. This discrepancy between the effect of the pandemic on depression versus anxiety may be explained by differences in its dynamics. That is, others have shown that the COVID-19 pandemic initially led to a short-term increase in anxiety, followed by a longer-lasting decrease in anxiety^40,41^, especially for people who initially had higher anxiety levels^42^. Since it took weeks to months to set-up this study, we may have missed the initial increase in anxiety around the onset of the pandemic.

A first strength of this study is the large sample size (*N* = 350). Online data collection allowed for repeated-measures in this large sample even during COVID-19 lockdown periods. Longitudinal data are the gold standard in resilience research^11^, since cross-sectional assessment might overestimate associations. Furthermore, we measured perceived stress and stressor exposure within the same period for all participants, which is rare in resilience research. This resulted in a reliable stressor-reactivity measure that could be compared between participants. Another strength is that we linked longitudinal survey data to pre-pandemic clinical measures in the same cohort, which were not yet influenced by the external stressor. Lastly, the subsample (*N* = 151) where two pre-COVID and one in-COVID visits were available, allowed us to control for the natural disease course.

The PPP cohort consists of patients with relatively early-stage PD, making it difficult to generalize results to the whole PD population. The sample was representative of the full PPP sample in terms of demographics (as shown in the Supplementary Table 2), but might have differed in COVID-19-related stressor exposure. Furthermore, the survey period only described a six-month period of the COVID-pandemic, whereas some long-term effects might arise later. Similarly, although we showed general progression in motor as well as psychiatric symptoms during follow-up, our follow-up period was up to two years, of which one year was during the COVID-19 pandemic, which might not be sufficient to show clear differences in motor progression trajectories between subgroups. Finally, pre-COVID measures were not measured directly before the start of the pandemic. It might be argued that low reactivity to stressors does not necessarily indicate resilience, given prior research showing that a diminished stress response to acute laboratory stressors correlates with poor mental outcomes^43–45^. However, while in these previous studies the *physiological* stress response was blunted, perceived stress levels were not^46^ - which is the measure we used here for quantifying SR. We assessed perceived stress repeatedly over a long time period of six months. Such longer-term stress responses are generally considered maladaptive, while acute stress responses are frequently adaptive^47^.

In conclusion, this study is one of the first to address resilience in PD, and it provides important insights on different levels. Resilience during the COVID-19 pandemic markedly differed between individuals, but was stable within individuals. Social support and non-motor symptoms (psychiatric and cognitive), rather than motor symptoms, predicted resilience in PD during the pandemic. This suggests that stressor-reactivity in PD is not predominantly determined by dopamine depletion, but may be associated with a more diffuse disease pattern^36^. These factors can be kept in mind during future public (or personal) health crises. Motor symptom progression was not accelerated during the pandemic and not influenced by stressor-reactivity, but depressive symptoms worsened specifically in stressor-reactive patients.

## Methods

### Participants

Participants of the PPP (disease duration at inclusion ≤5 years) were invited for this survey study^21^. The PPP study had an observation period of two years, with three annual in-person assessments at Radboudumc, Nijmegen, the Netherlands. For this study, we used data collected during clinical assessments (motor, cognitive and psychological tests).

### Ethics approval and informed consent procedure

All 520 PPP participants provided written informed consent during the first clinical PPP visit. All PPP participants included in April 2020 were invited by email to participate in this additional study. Subjects who were interested gave electronical informed consent (350/520 participants), in accordance with the Declaration of Helsinki. The Commissie Mensgebonden Onderzoek Region Arnhem-Nijmegen (reference number 2016–2934; NL59694.091.17) approved the study protocol and communication materials for the PPP study as well as the additional COVID-survey. The PPP is registered at ClinicalTrials.gov with registration number NCT03364894.

### Demographics and clinical data

Demographic data were available for all PPP participants. Clinical data that were collected during PPP visits and used for this study included time since PD diagnosis, medication use, comorbidities, Unified Parkinson’s Disease Rating Scale (MDS-UPDRS), Beck Depression Inventory (BDI), State-Trait Anxiety Inventory (STAI), Montreal Cognitive Assessment (MoCA), and Parkinson’s Disease Questionnaire (PDQ-39) to measure quality of life.

### Survey design

We used a repeated measures design with surveys at eleven timepoints within a six-month period (April-October 2020) during the COVID-19 pandemic (Fig. 1). Surveys were completed online using CastorEDC. At baseline (T1) and after six months (T11), a comprehensive survey was administered. In between, we conducted six (short) biweekly assessments during the first three months (T2-7), followed by three monthly assessments (T8-10). This resulted in up to eleven data points per participant.

T1 and T11 included measures of rumination (RRS^48^), perceived social support (SOZU-K^49^), and an early version of the positive appraisal style scale, process-focused (PASS-process^50^), which was used in other COVID-19-related resilience surveys^22^. Results of T1 are described elsewhere^51^. All surveys (T1-11) included measures of perceived stress (PSS^52^), stressor exposure (list of 18 COVID-19-related stressors and their burden, based on a previous resilience study^22^), PD symptom severity (MDS-UPDRS-self (Ib+II)^53^), and episodic anxiety (PAS-subscale B^54^). We quantified COVID-19-related stressor exposure at each timepoint as the sum of reported stressors, weighted by the burden per stressor. Examples of stressors include having COVID-19 symptoms or loss of social contacts (complete list in^51^).

### Statistical analysis

To quantify how strongly an individual’s mental health reacted to COVID-19 stressors, we first linearly regressed perceived stress (indicating stress-related problems) on stressor exposure across all participants and timepoints, to obtain a normative reactivity (non-linear relationships were tested, but did not outperform linear model). At a given timepoint, a participant’s regression expresses residual stressor-reactivity (SR), that is, to what extent the person reacts more (positive SR) or less (negative SR) to stressor exposure than predicted based on the average^16,22^, during the two weeks preceding that timepoint (Fig. 2a). The advantage of the residualization approach is that it can be compared between individuals with different stressor exposure levels, as it corrects for stressor exposure. This resulted in up to eleven scores per individual over the survey period, reflecting SR over time (Fig. 2b). Someone showing low reactivity over longer times can be classified as comparatively resilient^18^.

We conducted a latent class trajectory analysis using R-package lcmm^55^, to explore whether the sample could be divided into meaningful heterogeneous subgroups (latent classes) with different longitudinal trajectories of stressor-reactivity. We determined the best random effect structure based on the residual profile, and compared different parameterized link functions to allow non-linear trajectories of resilience. We then compared models with 1-4 subgroups and determined the optimal model structure by balancing between 1) model adequacy (based on the lowest Bayesian Information Criterion (BIC) value); 2) meaningful latent classes (based on plots of mean trajectories per class, and the requirement for each class to contain ≥2% of the total population); and 3) sufficient discrimination power (average posterior probability of assignments (APPA) to each class above 70%)^56^. Note that using SR scores as a basis for trajectory modeling is different from conventional approaches that use raw mental health scores^57^. The advantage is that, by using the exposure-controlled SR metric, we considered potential between-subject differences in stressor exposure and can thus exclude that an individual’s assignment to a trajectory class results from such differences. Otherwise, membership for instance in a low-symptom trajectory class might trivially reflect less exposure, rather than a resilient response to the experienced stressors^16^.

Having obtained latent SR classes, we used mixed-effect models with R-package nlme^58^ to explore trajectories of other longitudinal measures for these classes. Mixed-effects models are robust in case of missing data, meaning that models were estimated using all available data, even if timepoints were missing. Weeks since baseline survey (T1) were used as timescale, and we included natural cubic splines for time with knots at May 11 (week 3 after T1), June 1 (week 6 after T1), July 1 (week 10 after T1), August 18 (week 17 after T1) and October 13 (week 25 after T1) in the model to allow non-linearity of the model when there were important changes in governmental COVID-19 regulations (for details see Fig. 1a). SR class (as determined with the latent class trajectory analysis) was added as fixed factor. We used random intercepts and adjusted models for age, sex and living situation, and included interaction terms of age, sex and SR class with time. Because we used non-linear terms, effect sizes and estimates cannot be readily interpreted across analyses and are therefore not reported. Instead, we report diversions of trajectories based on the models’ predicted values and confidence intervals using R-package ggeffects^59^, and visualized trajectories per SR trajectory class, for a person of mean age with other characteristics set to the most common level.

To explore resilience factors and risk factors for SR during the COVID-19 pandemic, elastic net regression was performed using R-package glmnet^60^ Elastic net is a regularized regression approach that combines the advantages of both Lasso and Ridge regression^61^. Thereby, it allows for handling high-dimensional data with correlated predictor variables, providing a robust approach for variable selection and prediction. Ridge regression shrinks β-coefficients to prevent overfitting, and deals with multicollinearity of predictors. Lasso reduces overfitting by selecting a subset of features, while reducing coefficients of other features to zero.

As pre-processing steps, we dummy-coded all categorical variables, applied a zero-variance filter to potentially remove unbalanced variables, improved normality by applying a Yeo-Johnson transformation and z-scored all variables. We chose a fixed mixture (α) of 0.5, meaning that these two methods were equally represented, and determined the optimal penalty term (λ) between 0-3 by 10-fold cross-validation. We performed 1000 permutation tests to estimate the significance of the coefficients. This resulted in a subset of variables that predicted SR, and minimized the risk of overfitting while maximizing generalizability. Given the small changes in SR during the survey period, the average SR per participant was used as a continuous dependent variable, to allow for more variability and a more sensitive prediction model. We included 17 variables of interest (Table 2), which were all significantly associated with SR, as checked with univariate models for each predictor separately. We included 319 patients, due to missing values in one or multiple variables for 31 participants.

**Table 2:** All variables included in the elastic net regression analysis

Variables included in the elastic net regression		
Pre-COVID demographics	Pre-COVID clinical	Start COVID-pandemic
Age	PD disease duration	Perceived social support (SoZU)
Sex	Depressive symptoms (BDI)	Ruminative response (RRS)
Years of education	Anxiety (STAI)	Positive appraisal style (PASS)
Living situation	Cognitive impairment (MoCA)	
Main daily activity	Motor symptoms (MDS-UPDRS-III)	
	Non-motor symptoms (MDS-UPDRS-self)	
	Quality of life (PDQ-39)	
	Dopaminergic medication dosage (LEDD)	
	Comorbidities	

This table shows the 17 variables included in the elastic net regression. All of these were significantly associated (*p* < 0.005) with stressor reactivity, which was tested with separate univariate models for each predictor. The final prediction model contains a subset of these variables, while for other variables coefficients are reduced to zero.

To determine the effects of COVID-19 and participants’ mental health responses on PD symptom progression, we compared pre-pandemic MDS-UPDRS-III scores with in-pandemic scores one year later for the entire group with available complete data (*N* = 257). For the sake of simplicity, we compared the previously determined SR trajectory classes. Pre-pandemic scores were the last scores collected before March 11, 2020, when the World Health Organization declared COVID-19 a pandemic. A mixed ANCOVA was run to determine the effect of two independent factors, stressor-reactivity CLASS (low vs. high) and TIME (pre-COVID-19 vs. in-COVID-19), on the MDS-UPDRS-III, after controlling for disease duration, changes in levodopa equivalent daily dosage (LEDD) since the previous visit, age and sex (covariates). Patients with missing scores for either of the visits were excluded from this analysis (*N* = 66; visits were canceled due to COVID-19 lockdowns). Similar mixed model ANCOVA’s were used to test for the effect of the two same independent factors on depressive (BDI) and anxiety symptoms (STAI), while correcting for age, sex, and disease duration (covariates). We verified whether similar effects were present for the continuous SR, for which results are presented in Supplementary Note 2.

Data cleaning and analysis were performed in R-4.2.1 (www.r-project.org).

### Reporting summary

Further information on research design is available in the Nature Research Reporting Summary linked to this article.

### Supplementary information

Supplementary Material

Reporting summary

## Data Availability

The data that support the findings of this study are part of the Personalized Parkinson Project (PPP), and will be made publicly available upon the completion of the study. Data can be made directly available to qualified researchers upon request from the corresponding author. All participants provided informed consent for sharing of research data. The Research and Data Sharing Review Committee (RDSRC) will oversee the sharing of study data.

## Code availability

The underlying code that is used for the analysis of the data described in the current manuscript is publicly available at https://github.com/AnoukvanderHeide/Resilience_in_PD.
