# Author Correction: Predictors of stress resilience in Parkinson’s disease and associations with symptom progression

**DOI:** 10.1038/s41531-024-00718-x

**Published:** 2024-05-29

**Authors:** Anouk van der Heide, Lisanne J. Dommershuijsen, Lara M. C. Puhlmann, Raffael Kalisch, Bastiaan R. Bloem, Anne E. M. Speckens, Rick C. Helmich

**Affiliations:** 1 Radboud University Medical Centre, Department of Neurology, Centre of Expertise for Parkinson & Movement Disorders, Nijmegen, the Netherlands; 2 Radboud University, Donders Institute for Brain Cognition and Behavior, Centre for Cognitive Neuroimaging, Nijmegen, the Netherlands; 3 Leibniz Institute for Resilience Research, Mainz, Germany; 4 Neuroimaging Center, Focus Program Translational Neuroscience, Johannes Gutenberg University Medical Center, Mainz, Germany; 5 Radboud University Medical Centre, Department of Psychiatry, Nijmegen, the Netherlands

**Keywords:** Parkinson's disease, Epidemiology, Risk factors, Predictive markers

Correction to: *npj Parkinson’s Disease* (2024) **10**:81; 10.1038/s41531-024-00692-4, published online 11 April 2024

In the Acknowledgments section, funding from European Union’s Horizon 2020 research and innovation program and Grant Agreement number 777084 (DynaMORE project) were omitted. The original article has been corrected.

