# Supplementary material for: Predictors of stress resilience in Parkinson’s disease and associations with symptom progression
Source: NPJ Parkinsons Dis. 2024 Apr 11;10:81. doi: 10.1038/s41531-024-00692-4 (PMC11009258; doi:10.1038/s41531-024-00692-4)
Supplement: Supplementary file 1 — Supplementary Material [file 41531_2024_692_MOESM1_ESM.docx]

Supplementary material

**Supplementary Note 1: Mediation analysis**

The above elastic net regression highlighted PAS as an important resilience factor for SR (Figure 3 in the main text). According to the Positive Appraisal Style Theory of Resilience (PASTOR^1^), positive appraisal style is a resilience mechanism that may constitute a common pathway from resilience factors such as social support to resilience as an outcome. To test this hypothesis, in a follow-up analysis, we attempted to replicate previous evidence that PAS acts as a mediator for perceived social support (e.g. ^2^ and ^3^). Mediation analyses were conducted following a Baron and Kenny approach^4^ and the distribution-of-the-product method was used to determine indirect paths (see ^2,3^) in linear models controlling for age, sex and years of education.

We could identify a significant mediation of the effect of perceived social support on average SR scores via greater PAS (estimated mediated effect: -0.45, p<.001; 95% CIs: -0.73 to -0.22 at alpha = 0.001) in the present PD patient population. Compared to previous evidence for PAS mediation, which was found for example in a large cross-sectional sample^2^ and a smaller longitudinal^3^ from the general population, this suggests generalizability of the meditation pattern across populations with different disorder statuses.

**References:**

1 Kalisch, R., Muller, M. B. & Tuscher, O. A conceptual framework for the neurobiological study of resilience. *Behav Brain Sci* **38**, e92, doi:10.1017/S0140525X1400082X (2015).

2 Veer, I. M. *et al.* Psycho-social factors associated with mental resilience in the Corona lockdown. *Transl Psychiatry* **11**, 67, doi:10.1038/s41398-020-01150-4 (2021).

3 Bögemann, S. *et al.* Psychological resilience factors and their association with weekly stressor reactivity during the COVID-19 outbreak in Europe. *PsyArXiv*, doi:10.31234/osf.io/f7sy3 (2022).

4 Baron, R. M. & Kenny, D. A. The moderator-mediator variable distinction in social psychological research: conceptual, strategic, and statistical considerations. *J Pers Soc Psychol* **51**, 1173-1182, doi:10.1037//0022-3514.51.6.1173 (1986).

**Supplementary Note 2: Effect of the COVID-19 pandemic on PD for continuous measure of SR**

We validated our results regarding the effect of the COVID-19 pandemic (pre-COVID vs. in-COVID; one-year follow up period) on PD symptoms per stressor-reactivity (SR) class, by running linear mixed models for our continuous measure of SR. We only performed this additional analysis for the complete sample (last pre-COVID visit vs. first in-COVID visit).

*Motor symptoms*: MDS-UPDRS-III scores did not increase more rapidly when SR scores were higher (no TIME*SR interaction), but symptoms generally worsened over the course of one year (main effect of TIME: F(1,256)=8.7, p=.004) and motor symptom severity was higher for people with higher SR (main effect of SR: F(1,252)=13.0, p<.001).

*Depressive symptoms*: BDI scores increased during the one-year period, and this increase was more substantial for patients with higher SR scores (TIME*SR interaction: (F(1,237)=6.14, p=.014).

*Anxiety symptoms:* STAI scores were much higher for people with higher SR (main effect of SR: F(1,235)=174.3, p<.001), but over the one-year period in which the pandemic started, STAI scores did not change (main effect of TIME: F(1,238)=3.96, p=.491). Differences in SR scores did not affect a possible change in STAI scores over time (no TIME*SR interaction).

**Supplementary Table 1. Summary of elastic net regression results**

| **Best fit model: R^2^ = 0.35, standard error = 0.0046, *p*<.001**  α = 0.50 (fixed), λ = 0.51 (determined by 10-fold cross-validation) | |
| --- | --- |
| **Predictor** | **Regression coefficients (β)** |
| Anxiety (STAI) | 1.75 |
| Ruminative response (RRS) | 0.76 |
| Non-motor symptoms (MDS-UPDRS-self) | 0.34 |
| Living with family | 0.16 |
| Presence of comorbidities | 0.13 |
| Disease duration (years) | -0.02 |
| Age (years) | -0.04 |
| Perceived social support (SoZU) | -0.36 |
| Positive appraisal style (PASS) | -0.37 |
| Cognitive impairment (MoCA) | -0.42 |
| Quality of life (1-PDQ) | -0.68 |

Regression coefficients (β) of elastic net analysis for predicting stressor-reactivity during the COVID-19 pandemic in PD. Negative predictors qualify as resilience factors, positive predictors qualify as risk factors. Remaining variables were not included in the best fit model (i.e.) coefficients were reduced to zero).

**Supplementary Table 2. Comparison COVID-survey responders and non-responders of PPP sample**

| Variable | Survey sample (*N*=350) | Whole PPP (*N*=520) | Responders vs. non-responders |
| --- | --- | --- | --- |
| Demographics (pre-COVID) | | | |
| Age, years (SD) | 62.8 (9.0) | 62.8 (9.2) | p=.643 |
| Sex, *N* (% women) | 134 (38.3%) | 214 (41.2%) | p=.082 |
| Education, years (SD) | 17.4 (4.0) | 17.2 (4.2) | p=.176 |
| Living situation, N (%)  With partner  With family  Alone  Nursery home | 226 (64.6%)  90 (25.7%)  34 (9.7%)  0 (0.0%) | 340 (65.9%)  120 (23.3%)  49 (9.5%)  1 (0.0%) | p=.362 |
| Main daily activity, *N* (%)  Retired or pre-retirement  Paid job  No paid work  Voluntary work  No paid work due to illness  Not working  Household duties  Involuntarily no job | 143 (40.9%)  107 (30.6%)  22 (6.3%)  56 (16.0%)  19 (5.4%)  3 (0.9%) | 208 (40.3%)  148 (28.7%)  34 ( 4.7%)  86 (16.7%)  34 ( 6.6%)  6 ( 1.2%) | p=.419 |
| General disease characteristics (pre-COVID) | | | |
| PD duration, years (SD) | 3.7 (1.7) | 3.8 (1.9) | p=.596 |
| Levodopa equivalent dose, mg/day (SD) | 563.8 (368.6) | 585.8 (365.2) | p=.004* |
| MDS-UPDRS-III, total (SD)  OFF PD medication  ON PD medication (*N*=330) | 34.8 (12.8)  29.6 (12.5) | 34.8 (12.9)  30.0 (12.7) | p=.869  p=.262 |

This table shows mean (SD) or percentages for demographic and some general clinical characteristics (disease duration, medication dosage and motor symptom severity) for PPP participants that responded to the COVID-19 survey study (column 1), and for the whole PPP sample (column 2). Column 3 shows p-values of differences between invited non-responders and all responders, which was tested with t-tests for continuous variables measured and with χ^2^-tests for categorical variables. We indicated significant differences of p<.05 with *.
