# Supplementary material for: Predictors of stress resilience in Parkinson’s disease and associations with symptom progression
Source: NPJ Parkinsons Dis. 2024 Apr 11;10:81. doi: 10.1038/s41531-024-00692-4 (PMC11009258; doi:10.1038/s41531-024-00692-4)
Supplement: Supplementary file 2 — Reporting summary [file 41531_2024_692_MOESM2_ESM.pdf]

nature research

Corresponding author(s): Anouk van der Heide (NPJPARKD-02205R)

Last updated by author(s): Feb 16, 2024

Reporting Summary

Nature Research wishes to improve the reproducibility of the work that we publish. This form provides structure for consistency and transparency in reporting. For further information on Nature Research policies, see our Editorial Policies and the Editorial Policy Checklist.

Statistics

For all statistical analyses, confirm that the following items are present in the figure legend, table legend, main text, or Methods section.

n/a	Confirmed	
☐	☒	The exact sample size (n) for each experimental group/condition, given as a discrete number and unit of measurement
☐	☒	A statement on whether measurements were taken from distinct samples or whether the same sample was measured repeatedly
☐	☒	The statistical test(s) used AND whether they are one- or two-sided<br>*Only common tests should be described solely by name; describe more complex techniques in the Methods section.*
☐	☒	A description of all covariates tested
☐	☒	A description of any assumptions or corrections, such as tests of normality and adjustment for multiple comparisons
☐	☒	A full description of the statistical parameters including central tendency (e.g. means) or other basic estimates (e.g. regression coefficient) AND variation (e.g. standard deviation) or associated estimates of uncertainty (e.g. confidence intervals)
☐	☒	For null hypothesis testing, the test statistic (e.g. F, t, r) with confidence intervals, effect sizes, degrees of freedom and P value noted<br>*Give P values as exact values whenever suitable.*
☒	☐	For Bayesian analysis, information on the choice of priors and Markov chain Monte Carlo settings
☒	☐	For hierarchical and complex designs, identification of the appropriate level for tests and full reporting of outcomes
☐	☒	Estimates of effect sizes (e.g. Cohen's d, Pearson's r), indicating how they were calculated

Our web collection on statistics for biologists contains articles on many of the points above.

Software and code

Policy information about availability of computer code

Data collection	We used Castor (https://data.castoredc.com) to collect survey data. All data were stored with polymorphic encryptions and pseudonyms (PEP), to guarantee the privacy of participants but also to enable data sharing with interested researchers. This system allows qualified researchers to have access to the entire dataset or a subset of the dataset with specific keys for decryption.
Data analysis	We used R-4.2.1 for data analysis. Most important R-packaged that were used, are lcmm, nlme, ggeffects, and glmnet. The underlying code that is used for the analysis of the data described in the current manuscript is publicly available at https://github.com/AnoukvanderHeide/Resilience_in_PD.

For manuscripts utilizing custom algorithms or software that are central to the research but not yet described in published literature, software must be made available to editors and reviewers. We strongly encourage code deposition in a community repository (e.g. GitHub). See the Nature Research guidelines for submitting code & software for further information.

Data

Policy information about availability of data

All manuscripts must include a data availability statement. This statement should provide the following information, where applicable:

- Accession codes, unique identifiers, or web links for publicly available datasets
- A list of figures that have associated raw data
- A description of any restrictions on data availability

The data that support the findings of this study are part of the Personalized Parkinson Project (PPP), and will be made publicly available upon the completion of the study. Data can be made directly available to qualified researchers upon request from the corresponding author. All participants provided informed consent for sharing of research data. The Research and Data Sharing Review Committee (RDSRC) will oversee the sharing of study data.

Field-specific reporting

Please select the one below that is the best fit for your research. If you are not sure, read the appropriate sections before making your selection.

☐ Life sciences ☒ Behavioural & social sciences ☐ Ecological, evolutionary & environmental sciences

For a reference copy of the document with all sections, see nature.com/documents/nr-reporting-summary-flat.pdf

Behavioural & social sciences study design

All studies must disclose on these points even when the disclosure is negative.

Study description	We used a repeated measures design with surveys at eleven timepoints within a six-month period (April-October 2020) during the COVID-19 pandemic.
Research sample	520 participants of the PPP (disease duration at inclusion ≤5 years) were invited for this survey study, of whom 350 were included. The PPP study had an observation period of two years, with three annual in-person assessments at Radboudumc, Nijmegen, the Netherlands. For this study we used PPP data collected during clinical assessments (motor, cognitive and psychological tests), in addition to collected survey responses during the COVID-pandemic.
Sampling strategy	No sample size calculation was performed: the study was exploratory and used a sub-sample of participants from a large cohort study. All PPP participants were invited, and if interested informed about the content of the COVID sub-study. After receiving all study information, participants affirmed an online statement of informed consent.
Data collection	All survey data was collected online. Clinical data that were used, were collected during annual study visits, by researchers that were not involved in analyzing the data.
Timing	Data collection for this survey study took place between April 2020 and October 2020.
Data exclusions	Only one participant was excluded from data analysis, because this person did not complete any of the surveys after agreeing to participate in the study.
Non-participation	Participants in the PPP cohort could choose whether they wanted to participate in this additional survey study. They did not have to provide a reason for not participating. 350 of the 520 invited PPP participants were included in this additional COVID-survey study (67%). In Supplementary table 2, we compare PPP participants that responded to the COVID-19 survey study with the whole PPP sample and did not observe differences regarding demographic or clinical variables.
Randomization	N.A.

Reporting for specific materials, systems and methods

We require information from authors about some types of materials, experimental systems and methods used in many studies. Here, indicate whether each material, system or method listed is relevant to your study. If you are not sure if a list item applies to your research, read the appropriate section before selecting a response.

Materials & experimental systems

n/a	Involved in the study	
☒	☐	Antibodies
☒	☐	Eukaryotic cell lines
☒	☐	Palaeontology and archaeology
☒	☐	Animals and other organisms
☐	☒	Human research participants
☒	☐	Clinical data
☒	☐	Dual use research of concern

Methods

n/a	Involved in the study	
☒	☐	ChIP-seq
☒	☐	Flow cytometry
☒	☐	MRI-based neuroimaging

Human research participants

Policy information about studies involving human research participants

Population characteristics	350 participants were included (38.4% women), with a mean (SD) age of 62.7 (9.0) years at the baseline survey. The mean PD disease duration was 3.8 (1.6) years.
Recruitment	All PPP participants included in April 2020 were invited by email to participate in this additional study. Subjects who were interested received the participant information, after which they gave electronical informed consent.

Ethics oversight	The Commissie Mensgebonden Onderzoek Region Arnhem-Nijmegen (reference number 2016–2934; NL59694.091.17) approved the study protocol and communication materials for the PPP study as well as the additional COVID-survey. The PPP is registered at ClinicalTrials.gov with registration number NCT03364894.

Note that full information on the approval of the study protocol must also be provided in the manuscript.
